# Oral Tradition as Context for Learning Music From 4E Cognition Compared With Literacy Cultures. Case Studies of Flamenco Guitar Apprenticeship

**DOI:** 10.3389/fpsyg.2022.733615

**Published:** 2022-04-29

**Authors:** Amalia Casas-Mas, Juan Ignacio Pozo, Ignacio Montero

**Affiliations:** ^1^Faculty of Education, Department of Didactics of Languages, Arts and Physical Education, Complutense University of Madrid, Madrid, Spain; ^2^Faculty of Psychology, Department of Basic Psychology, Autonomous University of Madrid, Madrid, Spain; ^3^Faculty of Psychology, Department of Social Psychology and Methodology, Autonomous University of Madrid, Madrid, Spain

**Keywords:** oral learning, instrumental learning, embodiment, discourse about practice, musical practice, flamenco culture of learning

## Abstract

The awareness of the last 20 years about embodied cognition is directing multidisciplinary attention to the musical domain and impacting psychological research approaches from the 4E (embodied, embedded, enactive, and extended) cognition. Based on previous research regarding musical teaching and learning conceptions of 30 young guitar apprentices of advanced level in three learning cultures: Western classical, jazz, and flamenco of oral tradition, two participants of flamenco with polarised profiles of learning (reproductive and transformative) were selected as instrumental cases for a prospective *ex post facto* design. Discourse and practice of the two flamenco guitarists were analysed in-depth to describe bodily issues and verbal discourse on the learning practice in their natural contexts. Qualitative analysis is performed on the posture, gestures, verbal discourse, and musical practice of the participants through the System for the Analysis of Music Teaching and Learning Practices (SAPIL). The results are organised attending: (a) the *Embodied* mind through differential postures and gestures of flamenco participants that showed a fusion among verbal, body language, and musical discourse with respect to the musical literacy cultures; (b) the *Embedded* mind and a detailed description of circumstances and relationships of the two flamenco participants, and how music is embedded in their way of life, family and social context, and therefore transcends musical activity itself; (c) the *Enactive* mind, regarding the active processes that make differences between the reproductive and the transformative flamenco apprentices, then tentative relationship are observed in the discourse of each apprentice and the way in which they practice; finally, (d) the *Extended* mind through the bodily, technical and symbolic tools they use during learning. Flamenco culture of oral tradition made use of listening, and temporary external representations instead of notational, but also the body played a central role in a holistic rhythm processing through multimodality, such as singing, playing, and dancing. Conclusions point out the embodied mind as a result of the culture of learning reflected through the body and the gesture in instrumental learning.

## Introduction

Traditionally, analyses on musical apprenticeship processes began from academia and were mostly focused on the acquisition and transfer of Western European classical music. As soon as apprenticeship learning processes began to be studied in popular and traditional music cultures multifactorial nuances and differences were observed. These factors had repercussions on the activation of different mechanisms by the apprentices which at times involved a different focus or approach to the music. Often the new apprenticeship cultures studied, which did not have to be actually new (Leung, 2018), were described as “otherness” or cultural alterity, i.e., in reference to the previously defined standard of the academic area. Otherness is an epistemological stance that discursively explores the image of cultures that built up their space in the periphery or other intermediate cultural spaces (Sosa, 2009). It is a type of analysis that works as a discourse in specific historic contexts and is deployed in the social, legislative, institutional, and material order through processes that gain meaning through language and in this study through gesture and practice as well.

We cannot simplify or reduce the idea that formal music learning is synonymous with Western classical music using scores, and that informal learning is restricted to popular music transmitted by ear (Middleton, 1981; Folkestad, 2006). What is learned and how the elements are interconnected are not shaped by the type of music itself, but rather by a given approach to music that uses certain tools (representations, processes, contents, and conditions) as mediators for people developing within a *milieu*. These approaches are what we call learning cultures (Olson and Bruner, 1996), defined as the set of shared beliefs and practices on how to organise and promote learning, in this case, musical learning. We approach them without placing anyone above the other, simply directing our attention to elements that may not have been previously taken into consideration. Thanks to previous research on popular music (Green, 2002, 2008; Shah, 2006) educational spheres shifted the focus from teaching to learning, and thus from teacher to apprentice (Casas-Mas, 2022). In addition, today they provide us with a series of analysis categories in which differences are established with respect to formal realms, such as goals, planning, participants, motivation, place, learning style, leadership, and intentionality. But we still need to develop what that sociocultural construction affects in the embodied minds of the members of these communities or environments.

This research aims to provide a novel and therefore exploratory qualitative approach due to being an investigation that delves into the body, the gesture, the posture, the musical production, and the verbal discourse without antecedents, and also comparing these aspects for the first time to cultures with and without musical literacy. Firstly, a comparative disclosure of bodily and gestural issues is performed in three cultures of music learning and then to justify and make an in-depth description of the elements that characterise flamenco guitar learners in the context of the oral tradition. Our approach uses immersion and follow-up processes of students and their environments, in order to describe the culture from the words and gestures of the participants. This is achieved through their personal communicative processes, being treated with the greatest respect, and always monitoring the type of observation and interview.

For this reason, we begin by profiling the framework of 4E (embodied, embedded, enactive, and extended) cognition in relation to music, and then the relations between discourse and practice, or the differences of verbal and action productions. We will then present some nuances through reproductive and transformative learning conceptions regarding different cultures of learning music and the external representations or those “signs” each culture uses to either communicate or self-regulate in apprenticeship and that provide us with vital clues on the construction that could occur on a cognitive, emotional and corporal level. Finally, we will explain several common ideas about the culture of flamenco learning in communities of oral tradition; a non-uniform culture, but with some common music learning strategies because explicit forms of music notation, such as sheet music, are not used. We are interested in the connection with the body, sounds, and gestures that help us understand the experience and externalisation of music from a perspective not yet investigated in-depth within flamenco culture. Cognition 4E help to understand the social aspects of music, particularly those found on the oral transmission, since the body plays its part through action where verbal discourse does not predominate, and the body is an extension of the mind (or vice versa), and even the musical instrument is also an added extension (Nijs, 2017; Schiavio and van der Schyff, 2018).

### Embodied, Embedded, Enactive, and Extended Cognition and Musical Demeanour

The dualism of the classic computational models that depicted both psychological research and education were characterised by splitting the mind and the body, prioritising the first and leaving as a second category those learnings coming from psychomotor action, movement, emotion, and ignoring the specific context in which they are inserted (Claxton, 2015; Pozo, 2017). The philosophical roots on which this approach is based have been highly questioned in the last three decades until the statement that not only the mind is in the body but that the body is also in the mind (Varela et al., 1991; Damasio, 1994). The processes that the musical domain activates in human beings are already an embodied form of action that we adopted to make sense of certain properties of the environment that are discovered in a deep continuity between cognitive and biological processes (Maturana and Varela, 1980; Varela et al., 1991; Schiavio et al., 2017). If we assume some principles of a deep continuity between cognitive and biological processes, through feedback processes of imitation (Donald, 1991; Mithen, 2005), then a dualistic division closes the possibilities of understanding a multimodal and holistic phenomenon as music.

In the analytical effort of science there are authors who divide the notion of the body in two; on the one hand the physical, measurable, and “objective” body, and on the other the body from the phenomenological point of view, which occupies the metabolic, emotional, and other aspects (Schiavio and van der Schyff, 2018). To say that cognition is *Embodied* means for them that our mental life depends directly on these two (objective and phenomenological) descriptions of the body. For example, the student who is learning to play the violin can become aware of his bodily representations if he records himself while playing (his body tension, the relationship between the movement of the bow and the sound obtained, etc.) (Pozo et al., 2020b). This in turn feeds back into the neurological activity, because it plays an important role in training the musician’s physical abilities and thus developing his sense of himself as a skilled and embodied agent (Høffding and Schiavio, 2021).

That new 4E mind (Rowlands, 2010; Nagy, 2017) would not only be *Embodied*, but also *Enactive*, or based on action rather than on the contemplation of the world (e.g., Bowman, 2004; Pozo, 2017; Schiavio et al., 2020). In the same example above, the actual action performed, even mimetic, and only emulated without the instrument (Pozo et al., 2020b), provides the essential content of the musical representation that links sound and action. Students learn better when they play music instead of seeing someone else playing it (Schiavio and van der Schyff, 2018). An *Embedded* cognition is due to it being always situated in an environment from which it cannot be separated without losing its meaning. The learning and production of music cannot be separated from the cultural context in which it is produced. As already mentioned, various studies have shown that music is conceived, lived, and felt differently in the contexts of formal and informal education (Green, 2002; Casas-Mas et al., 2015a).

Finally, Newen et al. (2018) defend that a cognitive process is strongly embodied by bodily processes if it is essentially based on processes in the body that are not in the brain, or is strongly embodied by extra-bodily processes if it is partially constituted by extra-bodily processes. In the example mentioned before, when the student records himself to analyse his bodily actions and their relationship with the sound achieved, the video constitutes an external representation from which to redescribe his own action (Pozo et al., 2020b). At a different level, the scores constitute a resource that allows the performer to reconstruct their own bodily action; such as the sequence of actions to perform and fingering (Casas and Pozo, 2008). Thus, the mind is *Extended* since the mental action would be expanded beyond the brain, not just to the rest of the body, but to the extracorporeal material and symbolic resources in which it is supported. The mind emerges in relation to devices and environments that co-constitute music-like behaviours, and not only “afford” them (Clark and Chalmers, 1998; Schiavio et al., 2017).

### Coherence Between Discourse and Practice

The social and communicative aspects of music, especially through the oral transmission learning and extralinguistic elements could be a priority research objective in music due to the repercussion they have on the body and the musical instrument as extensions and feedback of the mind (Nijs, 2017; Schiavio and van der Schyff, 2018). The music fits into movement, dialectic, emotion, and shared experiences with other beings because it is an inherently social phenomenon and this is precisely what lends it meaning (Cross, 2003). Nevertheless, a new division between the terms of theory and practice appears, between what beings report and what they do in reality, and this begins with the ways in which we approach the phenomenon from research.

Traditionally, the study of conceptions and approaches to learning music were based on verbal information uptake (e.g., López-Íñiguez and Pozo, 2014; Marín et al., 2012), either showing interest in finding out what musicians focus their attention on during the different stages of studying and practicing sheet music (Williamon et al., 2002; Chaffin et al., 2003) or without a score (Green, 2002, 2008). Recently practices are gaining greater emphasis (López-Íñiguez and Pozo, 2016; Boucher et al., 2020), because they inform us about elements that the *Embodied* mind may be hiding behind verbal speech, in addition to appearing to be going at a different speed compared to what a person expresses verbally. Studies of analysis regarding consistency between discourse (as verbal productions) and practice (as sequence and structure of actions) reveal the existence of a major gap in the area of instruction (Torrado, 2006; Clarà and Mauri, 2010; Buehl and Beck, 2015; Clarà, 2019; Úcar, 2021) where verbal expression is higher in complexity than teaching practices. Pozo et al. (2006) observe a frequently produced dissociation between implicit embodied representations of teaching and learning (the somatosensory states associated with physical actions or emotions) and our explicit knowledge of them (the attributions or explanations that we give ourselves over the reasons for these often biased emotional states or physical actions). Examples of research and intervention proposals are focused on developing metaphoric experiences (Zbikowski, 2015; Gibbs, 2017) and reflection on practice (Bain et al., 2002). In relation to musical learning, it could be possible that the coherence between verbal discourse and body language may depend more on the conceptions of learning, for example, reproductive versus transformative, as different types of *Enactive* mind, rather than on the culture of musical learning (Casas-Mas, 2020).

### Reproductive and Transformative Learning in Music

The premise of cultural preservation could easily be related to the context of oral tradition as a necessity for preserving the passing on of information. However, this also occurs in traditions with highly developed literacy, such as the Western European classical music learning culture, precisely because it shares a mode of production with flamenco (Pozo et al., 2022a) in which the piece reaches the apprentice as closed, and the apprentice is expected to “correctly” reproduce it. In the former, the piece reaches the apprentice as closed through the musical score and mediation of the teacher and in the flamenco of oral tradition mediating by the teacher through the oral-procedural tradition. This differs from learning cultures where the pieces reach the apprentice and they have to complete them either individually or collectively through improvisation, such as in jazz (Murphy, 1994; Gioia, 1997).

Different modes of music production refer to the internal structure that every group learning situation possesses and that organises social interaction during practice (Tagg, 1982; Kagan, 1989). The musical production is the process that transforms a hypothetical silence into a complete cultural product (Baño, 2018). The Western European classical and flamenco cultures of oral tradition are opposite in the formal-informal continuum, but have similar modes of production, because the piece is closed to the learner, either because of the score or because of the teacher (Baño, 2018; Torrado and Mengual, 2020). Therefore, the purpose of instruction tends to different reproduction degrees of the piece. On the other hand, in the jazz culture of learning the mode of production is more open and ends through the person or group that improvises (Murphy, 1994; Gioia, 1997; Hickey, 2015), in our case the apprentices. This is an example where reproduction of the theme is not a priority but the use of characteristic elements of the language or style.

That said, the open or closed production model has many nuances, depending on the restrictions that the individual or group context imposes, through the teaching and learning conceptions. Put in another way: this is why within the same culture we may find more reproductive or more transformative apprenticeship processes. In the reproductive approach the apprentice is limited to faithfully copying the notational material, whilst in the transformative approach – also called exploratory – the apprentice needs to integrate the information. This difference was established by Scardamalia and Bereiter (1987) in relation to written language, which was later applied to the field of classical music and musical scores (Cantwell and Millard, 1994; Hallam, 1995; Hultberg, 2002). More superficial approaches to the learning of scores are shown here, in contrast to approaches that required greater autonomy and reflection by the apprentice to integrate the information from different sources.

In previous studies these apprenticeship polarities have been classified as direct and constructive (e.g., Bautista et al., 2010; López-Íñiguez and Pozo, 2014). The direct conception starts from a realistic epistemology in which knowledge reflects the object with fidelity, although with varying degrees of completeness or exhaustiveness. There is partial and complete knowledge. A linear and direct relationship is established between certain conditions (age, motivation, amount of practice, etc.) and the learning outcomes. Instead, the constructive conception implies a qualitative change because it is based on a relativistic epistemology in which knowledge is a construction elaborated in a social and cultural context in relation to certain goals. That construction provides tentative and alternative models for interpreting the object. Learning is understood from complex relationships between components that are part of a system. Nevertheless, in this study, it makes more sense to speak of either more reproductive or more transformative apprenticeships respectively because within the flamenco learning culture of oral tradition no comparable constructive profile has been found to that of other learning cultures. Furthermore, no previous studies have described in detail the possible nuances that could arise within the oral tradition of musical knowledge transmission.

Although classical and flamenco guitar traditions have common antecedents, such as the first guitar treatise played by Amat (1758) or the baroque and classical-romance guitar treatise (Torres, 2012), one singular element of flamenco guitar compared with the classical guitar is the fact that it continues requiring song and dance accompaniment training (Calahorro, 2019). This separates it from most academic musical practice, fostering the creativity and ability to adapt to the situation, that requires the development of intuition and spontaneity in musical production. Another element we believe could differentiate the academic tradition from the oral tradition is that the latter necessarily requires support in practice for its transmission. The former is based on musical notation and has therefore created verbal discourse through a process of development of elements and knowledge. This activates a type of music apprenticeship like a second language instead of like a mother tongue emanating from the oral-procedural tradition (Casas-Mas, 2018). As a result, the apprentice’s autonomy, their decision-making and their more or less reproductive use of material are key elements to take into consideration. These can be observed not only from their verbal discourse but also from their gestures, private singing, and how the learner uses the external representations (Casas-Mas et al., 2019). Therefore, the analysis needs to be made of these representations that conform to the *Extended* mind in the oral learning culture.

### External Representations in Music

Vygotsky (1933/1978), conceives of the human as a social and cultural being, and from here develops his theory on social training of the mind through the use of signs (or external representations). These are culturally created tools that are modified by human nature itself. External representations are those that express an international relationship between a series of signs with visual-spatial qualities but refer to another reality present or not, e.g., sound, thought, etc. (Martí and Pozo, 2000). Some are also deployed in a spatial dimension, e.g., notations based on (a) flexible combinations of rules typical of graphics, visual images, or designs on some surface, such as illustrations, maps, musicograms; and (b) the stricter notational systems, like alphabetical, metric or traditional musical scores, which remain outside of the content of this article. In the flamenco learning culture of oral tradition, we concentrate on those signs or external representations which are deployed temporarily and have a little structural definition, like the observable body and gesture representations and oral language, as classified by Pérez-Echeverría and Scheuer (2009). The *Extended* mind has been highlighted through gestures and mimesis (Goldin-Meadow, 2003) because they imply a different level of abstraction from notational systems since the gesture is always linked to the context of production. It is difficult to separate the gesture from the communicative or self-regulating situation that elicits it.

It is true that in the case of non-notational external representations, cognitive psychology has not yet dedicated much attention to describing and organising phenomena and we will base our theories on external representation studies and on research that analyses the self-regulating function of the self-referring gestures and private speech from which we will attempt to make our contribution. Private speech is an original Vygotskian concept defined as speaking to oneself for communication, self-guidance, and self-regulation of behaviour. It is neither intended for nor directed at others. Private speech is a crucial window providing insight into how language mediates and regulates thought processes (Lantolf and Thorne, 2006). However, we will make use of music for the wealth of signs or marks it can provide thanks to its historical role as a socio-cultural symbolic system, converting it into a tool for regulating social interactions and group behaviour (Livingstone and Thompson, 2009; Tarr et al., 2014). We take this concept to the musical field and see that the use of private singing, and vocal, guttural, whistling, etc., have also been observed, and we propose it has two main functions: (a) self-guidance and self-regulation, e.g., cultural tool in the regulation of a child’s behaviour in situations when they are falling asleep (Mead and Winsler, 2019), or (b) when learning a musical instrument (Casas-Mas et al., 2019), or managing waiting times (Winsler et al., 2011) with regard to the previous musical experience.

Thibodeaux et al. (2019) found a relationship albeit incipient, between the production of private singing and private speech. During tasks of selective attention when one appears, so does the other, with several similar self-regulating uses. There were no children who, when producing the first, in the form of song or humming did not produce the second, as speech in its overt form, as a self-guiding tool whilst carrying out a task. Even into adulthood, overt speech can be observed during challenging cognitive tasks (Duncan and Cheyne, 2001; Alarcón-Rubio et al., 2013). In general, we would expect children to move toward increased inner speech with age (Winsler et al., 2006), but this may depend on the type of task or area of executive function skill they are currently developing. It remains to be explored whether *private singing* (defined as a qualitatively self-regulatory and self-guidance tool involving some form of singing, guttural, or vocal sound) has a similar pattern. For the moment, it has been observed that in cultures that make use of explicit musical notation, such as scores (either traditional or in the form of jazz charts), greater use is made of private singing elements, some more explicitly, sonorous, and intelligible, while others more internalised, such as whispered or silent, guttural sounds, and verbal lip movements (Casas-Mas et al., 2019). The same authors indicate a more internalised use in popular musical cultures, like the one treated in this article, rather than in the academic ones.

### Flamenco Music, the Culture of Learning in Communities of Oral Tradition in Spain

Flamenco music is the product of cross-hybridisation when the traditional agrarian culture became transformed into today’s urban folklore, and popular religious festivals were transformed at the beginning of the 20th century into show business (Steingress, 2004). The initial reference to the existence of “new flamenco music” was made in 1853 (Sneeuw, 1991) in late nineteenth-century Paris. This was where the “dance schools” of the 18th century came to be called “Bolera School dances” (Junta de Andalucía, 2003) and involved an artistic re-interpretation of folk dances and theatrical repertoire of dances “on stage,” also at the grassroots level, with a greater professionalisation of performers. Here we are talking of flamenco music which like other popular Mediterranean types of music was defined as a piece of urban music at the beginning of the XX century. It has recently been acknowledged as the *Intangible Cultural Heritage of Humanity* by United Nations Educational, Scientific and Cultural Organization [UNESCO] (2010), but there are many aspects remaining, particularly urban flamenco, of its forms of production and transmission, which have still not been researched in Spain. This article analyses and attaches value to the recovery of the forms of flamenco apprenticeship within the communities in which it is orally transmitted.

The Flamenco culture of learning for instrumental learning had no formal academic accreditation in the country until very recently; it belonged to the oral tradition, and here we describe it to situate the *Embedded* mind in which its members develop. The incorporation of instrumental and vocal flamenco to academic studies in the Conservatory is very incipient in Spain. The Act of the regulation of the general educational system (Jefatura del Estado, 1990) was the first to mention, in Jefatura del Estado (1995), the specialty of flamenco and instrumental flamenco guitar and the Organic Law on Education (Jefatura del Estado, 2006) when a greater systematisation was carried out. Currently, the flamenco guitar at a tertiary educational level is poorly integrated into a few conservatories throughout the country, in cities, such as Córdoba, Murcia, Barcelona, Malaga, and recently, Jaen. Despite institutional neglect sometimes flamenco is now considered as a mark of identity in Spanish citizens of Roma ethnicity, despite the fact that there are many non-Roma professional flamenco musicians (Payos^1^). This article will spotlight the Roma ethnicity community in Madrid to reflect on apprenticeship issues which could also be present in non-Roma ethnicity communities in certain regions of Spain where flamenco music is acquired as a mother tongue.

The European Commission *Country Report Spain 2020* recognises that the Roma ethnicity community of today is inclined toward considerable social vulnerability. Only 17% of teenagers finish the first cycle of secondary education and 63% of Roma ethnic young people do not work, study or receive any training [European Unión Agency of Fundamental Rights [FRA], 2018; Roma Secretariat Foundation (FSG), 2019]. Early dropout from secondary education in Spain is high, at 16%, compared with the European average, at 14.9% (Ministry of Education and Professional Training, 2019), and more common than we would like to think in Roma communities, at 63.4% [Roma Secretariat Foundation (FSG), 2013]. García Fernández et al. (2017) performed an exhaustive review of primary and secondary school textbooks in Spain, concluding that the Roma population has been systematically omitted, and if it is mentioned, the tone is paternalistic toward or prejudiced against it. This has some improvements in the new 2022 Act of the regulation, that the Spanish government has established for Primary and Secondary Education. This type of omission has already been described in research studies with other communities (Lave, 2011; Karlsen, 2012; Sexton, 2012), and perhaps it is one of the factors to take into account in the high dropout rate.

Some authors have resonated this history of discrimination up to the present day [e.g., Casas-Mas, 2018; European Unión Agency of Fundamental Rights [FRA], 2018; Roma Secretariat Foundation (FSG), 2019], highly internalised in the culture. Even the Dictionary of the Royal Academy of the Spanish Language insists on maintaining the definition of Roma people as ‘‘trapacero’’ (sb. who with craftiness, falsehoods and lies attempts to trick someone in a matter), although at the request of the Roma community it incorporated in 2015 a usage note that warns against the meaning ‘‘as offensive or discriminatory’’^2^. Despite the conceptions and conditions surrounding this population, which is not monolithic, the Roma ethnicity community continues to attempt to strengthen its cultural cohesion ties that preserve some of the traits found in its origin. Its media are the preservation and replication of several values which are possibly regarded with bewilderment or disconcertion from “outside.” However, this reinforces and protects them from languages, ideas, policies, or behavioural patterns prevailing in their immediate environment.

This research study portrays two case studies of two participants belonging to the oral tradition of guitar learning in Roma ethnicity communities and whose speech and practice was remarkable from a previous sample of a multiple case study with guitarists from different learning cultures. The previous studies allowed us to hypothesise within this flamenco tradition and the main aim of this article is to probe into the contrast of this flamenco of oral tradition with other cultures of learning music regarding the body and gestures, and the learning differences within the same tradition. More specifically, we have formulated the following goals for this article:

1. First, to observe the peculiarities of guitar learners related to posture, gesture, and body, belonging to a flamenco learning culture of oral tradition, based on a previous comparative sample of Western classical, jazz, and flamenco guitar learners of oral tradition (Embodied mind).
2. Second, to describe the environment where these apprentices develop, taking into account the relations they establish with teachers, family, and peers (Embedded mind).
3. Third, to focus on observing if there are differences in the speech and learning practice between two flamenco guitarists selected as instrumental cases from the initial sample, with polarised approaches to learning, reproductive (R) and transformative (T), as differential active processes. The hypothesis is that intuitive conception about what it is to learn constitutes the starting point for the development of practice and action. We will observe whether or not there is coherence between the discourse and the practice of these learners (Enactive mind).
4. Finally, the observation of the forms and uses of external representations by the learners of oral tradition constitutes another of the main objectives of this study, because it provides a lot of information about the body, sound, and gesture, which has not been the objective of traditional research, more focused on verbal language. We examine such issues here which, to our knowledge, is the first article on this topic (Extended mind).

## Methods

In previous research, through the analysis of variance and the lexicometric analysis, three learning cultures were outlined as significantly different in their conceptions and verbal discourse about teaching and learning music (Casas-Mas et al., 2014, 2015a). It was carried out through a prospective *ex post facto* design, where the musical learning cultures of Western classical, flamenco, and jazz were representative of formal, informal, and non-formal educational environments, respectively (Trilla, 1997; Casas-Mas, 2013). Their teaching structure and intentionality made up the non-manipulable independent variable. Then, we set out to observe the discourse and practice of learners belonging to these cultures, which would be our dependent variables in the research. To highlight the nuances between forms of learning within each culture and not present them in a uniform way and as a single prototype, we selected the two participants with the most polarised conceptions and discourse (reproductive or transformative) from each of the three cultures. Thus, we implemented a multiple case study in which we observed and analysed their music learning practices in depth through the preparation of a piece and the situated discourse on practice. There were then six instrumental case studies (Stake, 1998) selected by the variable conception of learning within each culture of musical learning. This allowed us to observe the coherence between verbal discourse, discourse situated on musical practice, and practice (Clarà and Mauri, 2010). These flamenco case studies are drawn from this context to be illustrated in this article.

### Participants

The original sample was comprised of 30 guitar apprentices coming from Western classical, jazz, and flamenco of oral tradition as formal, non-formal, and informal cultures of learning, respectively, following Trilla (1997) and Green (2002). The categorisation of learning cultures covered: the degree and institutionalisation of teaching; the structure of the studies established in the national curriculum or not; whether they had teacher selection through competition or only merits; the regularity and intentionality of teaching, and the evaluation leading to accreditation. The Western classical culture of learning met all requirements of the formal realm. In the jazz culture of learning in Spain, there are learning structures, such as jam sessions, that break one or some of the formal realms which are essential to learner training, and they use chart-type musical scores that give a general notion of the piece beforehand. In the flamenco culture of oral tradition children from a very early age practice music (singing, dancing, playing) many hours in a family setting and with peers, during which they exchange exercises, *falsetas*, and pieces inherited from teachers or composed by themselves. Nowadays they often use video/audio recordings as tools, particularly on mobile phones, but they do not use any kind of music notation. There are no exams or specific accreditations.

The participants were guitarists at a semi-professional stage of learning who had educational and semi-professional experience in only one of the cultures of musical learning considered in this study. Based on this criterion, the sample is configured as follows: ten participants are from the classical culture of learning (5 men, 5 women), aged 19 to 29 years (*M* = 24.9; *SD* = 3.48), have spent more than 10 years studying music in formal realms, such as the conservatory, and are studying for a tertiary degree (equivalent to a Bachelor of Music degree) or master of music studies. They also have pre-university or university studies, and most of their families have some relationship with amateur music. Among the participants, ten are from the jazz culture of learning (9 men, 1 woman), aged 26 to 42 years (*M* = 29.6; *SD* = 4.93), with a college degree in non-musical studies, and most have studied for a professional degree in music (prior to tertiary studies). Their families have no relation to learning music. Furthermore, ten participants are from the flamenco culture of learning (10 men), aged 15 to 25 years (*M* = 16.82; *SD* = 2.96), all of Roma ethnicity, and most have not completed compulsory secondary education. They have been studying the guitar for 1 to 5 years with a specific teacher. There are professional musicians in all their families.

Considering the reproductive and transformative conceptions of learning, the two most opposite learners from each of the three cultures were selected to make an in-depth multiple case study with constant comparison analysis of their learning practice and their situated discourse about practice. After contrasting the hypothesis, some peculiar characteristics about teaching and learning in the oral tradition flamenco culture came to light, which showed it to be far removed from literate music cultures (Casas-Mas et al., 2014, 2015a). In this article, we will describe the two participants of the flamenco apprenticeship culture selected as instrumental case studies. The first is the apprentice with a more reproductive learning profile (hereinafter “R”) and the second with a more transformative profile (hereinafter “T”) in the Roma ethnicity communities observed.

### Materials and Procedure

#### Musical Piece

The pieces prepared by the two students were a *falseta*^3^ each by bulerías. Bulería is a fast flamenco rhythm (around 240 bpm) in 12 beats that may also be broken down into a measure of 6/8 followed by a measure of 3/4. It is most commonly in an A-Phrygian mode, with a sharpened third. It is traditional from Jerez de la Frontera, in Western Andalusia. Although on occasions it is played with *air* (speed) not excessively fast, its spirit is burlesque and playful. The twelve beats are usually those most indicative of flamenco, compared with other types of music; for this reason, we believe their inclusion to be most relevant, in addition to being those which the participants chose to work with. The *falseta* of the first student was from the maestro Víctor Monge, “Serranito,” based on a text by Federico García Lorca. The *falseta* of the second student was a composition by his teacher “El Viejín.”

#### Structured Interviews With Open-Ended Questions

An initial interview with each participant was conducted. This took a generic and global look at their learning expectations during the three learning phases of their musical piece: initial, intermediate, and final. After each of the three practice sessions (PS) a post-session interview was conducted, three in total, which was different from the initial one and was more detailed, where their opinion regarding learning from the previous session was sought and their expectations regarding the following PS (the interview protocol is included in the Supplementary Material).

#### Practice Sessions

We proceeded to record the PS of learning a piece of music for each apprentice. We recorded 3 PS for them: the first at the beginning of learning, the second after 2 weeks of preparation (in the middle), and the third when they were ready to play in public, around 3 weeks later, based on previous research (Nielsen, 1999; Williamon et al., 2002; Chaffin et al., 2003). We analysed the recordings of the PS in which the researcher also took notes *in situ*, to facilitate the appearance of private speech, and we fitted both pieces of information collected together.

#### Reflection on Practice Interview

We also combined verbal information collected and learning practice observation with reflection on practice interviews (RPI). In these, we watched the video of the PS with the apprentice, and we asked questions regarding points of interest from the research, and the apprentice also commented upon what was brought to his attention when watching his PS (interview protocol included in Supplementary Material). These interviews proved to be very rich in the information given by the apprentices and served to clarify thoughts, emotions, and decision-making with regards to what was observed by the researcher. The participants provided their written informed consent to participate in this study in accordance with the Regulations of the Research Ethics Committee of the Spanish university. Participants had the right to discontinue participation at any moment and were not compensated for their time.

This article is based on the System for the Analysis of Music Teaching and Learning Practice (SAPIL), which is widely developed in Pozo et al. (2022b). Categories are divided into three main sections: First, it deals with categories of learning conditions; that refers to the nature of the sociocultural environment that conditions the practice of learning from the human mediators that make it possible. Second, the processes of learning that include the explicitly or implicitly cognitive processes managed by the teachers and students, the cognitive demand of tasks, the level of the meta-cognitive management the students require of them, the memory or recovery strategies used. Finally, the results of learning or what is learned in a certain learning culture, what it values and encourages both explicitly and implicitly. The system contemplates the distinction between symbolic (where we include external representations), procedural, and attitudinal results of learning. The inter-reliability agreement is Fleiss’ Kappa > 0.8 for the system of analysis, SAPIL. In addition, we have carried out a triangulation to test validity through the convergence of information from different collecting information techniques: Structured Open-ended Interviews, Practice Sessions, Reflection on Practice Interviews, and triangulation with the apprentices at the same time.

## Results

The structure of the results section will be presented according to the 4E cognition; embodied, embedded, enacted, and extended. We begin by comparing the posture and gestures of the participants in the three cultures of learning as the *Embodied* mind, then we follow with the social environment and circumstances of the participants and a deep description of two flamencos, from the *Embedded* mind framework. In addition, a detailed description through microanalysis of practice and discourse about the practise of musical processes of learning, as *Enactive* mind. Finally, we analyse the use of external representations, but instead of a spatial notation system, we focus on the analysis of temporal ones, such as body and gesture and oral language for learning music as an *Extended* mind. These bodily, postural, and gestural categories are mentioned in this analysis system but are not specified because they are emerging from the results of this research. That is why we will begin and finish by explaining the results regarding them.

### Posture and Gestures in Three Cultures of Learning (Embodied Mind)

We will begin by describing the learning position with respect to the teacher, the posture, and the gestures that accompany the verbal discourse of these advanced guitar learners observed during the investigation. Teacher and student were positioned in an arc in the jazz and classical cultures of learning so they could both see the notational material, the score, located between them. Nevertheless, in the absence of notation communication between teacher and learner through body language was very important in flamenco, so the teacher was usually positioned opposite the student, who was accustomed to decoding the teacher’s movements from a contralateral position. The teacher’s eyes, eyebrows, shoulders, and head were involved in imbuing the musical content with significance, used very expressively for emphasising while accompanying musical performance.

**FIGURE 1:**
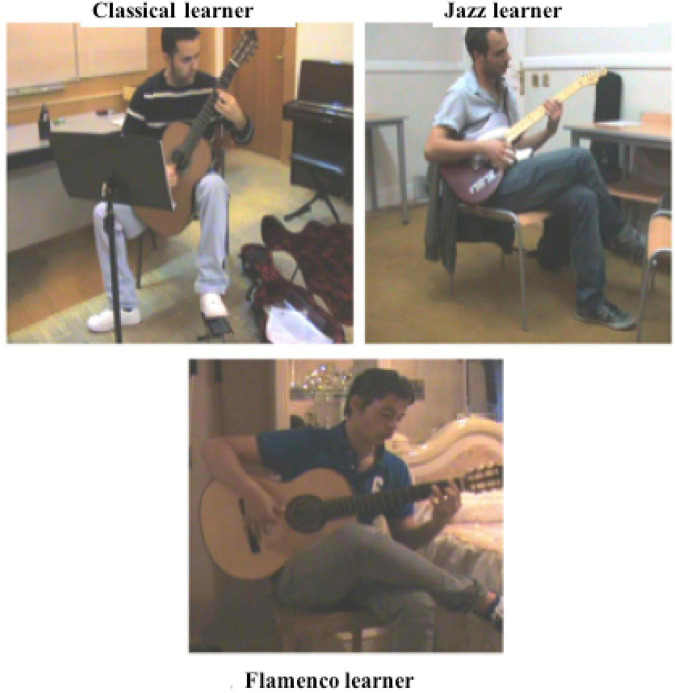
Learner posture during practice in three learning cultures.

The first issue to mention is that posture while playing an instrument is a relevant cultural acquisition that configures the embodied mind. We found different postures had been adopted in the three cultures during PS: the posture of classical participants could be defined as ergonomic, aimed at preventing pathologies due to lengthy exposure to practice (Carreras et al., 1996). The sitting posture of jazz participants looked relaxed and introspective, with legs completely crossed and the body leaning over the guitar as if drawing knowledge from the inside out. Flamenco participants’ posture was somewhere in-between: legs crossed, like jazz players, though in a more open position, with right ankle almost resting on left knee, and although they leaned over the guitar, their posture was more active than in jazz players, more similar to classical players. These three postures can be seen in Figure 1.

**FIGURE 2:**
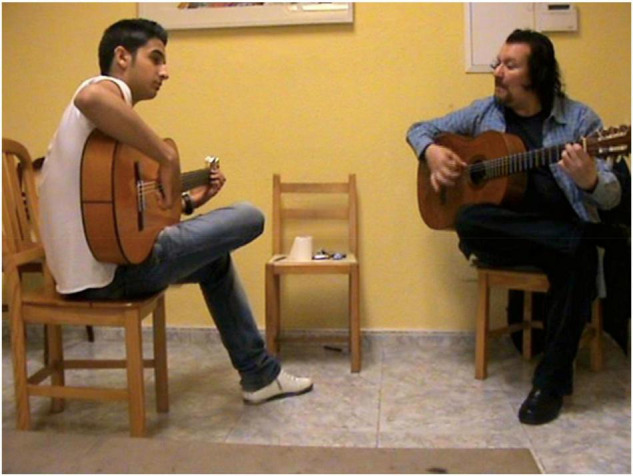
One-to–one class between the flamenco teacher Entri with student R, sitting opposite, with the teacher looking at the apprentice’s fretboard and the apprentice looking at the teacher’s fretboard.

Second, going into details regarding the distinctive position of apprentice and teacher in this flamenco of oral tradition culture, teacher and student sit opposite one another, so that the teacher’s guitar fretboard can be seen by the student sat opposite. This is not a mirror, it is so the student gets used to deciphering the teacher’s movements and performing them inversely from their own (Figure 2). The student is learning through what we could call ‘‘osmosis’’^4^, since they reproduce practically in real-time and simultaneously, the material they have not previously seen. We believe the importance of gestural communication and the teacher’s facial expressions is of great interest. The student usually watches the teacher’s guitar fretboard whilst reproducing what he plays, but the teacher mainly looks at the student’s eyes, as well as checking the student’s guitar fretboard from time to time.

The third and relevant question is the frequency of appearance of gestures that accompany verbal speech in these three learning cultures. Classical participants did not use the guitar while speaking but often gestured as if playing an *air guitar*. Jazz participants gave many examples of what they told us about, by playing them on the guitar, which they usually had nearby, but taking the guitar and leaving it in the case after each example. Flamenco participants displayed a fusion among verbal, body language, and musical discourse: their discourse was completed in the three forms while remaining incomplete in any of the three forms separately. They always held the guitar, making it sound constantly, without any exemplifying purpose. Sometimes they would replace the beginning of the end of a verbal phrase with a musical phrase on the guitar, in which the prosody usually matched the verbal language it was substituting (Supplementary Video 1 in Supplementary Material). Another example was the way in which flamenco participants stamped a foot to support what they were explaining in verbal discourse, as an important repeated gesture that was not used by apprentices in other cultures. A comparative frequency of gestures in these three cultures of learning can be observed in Table 1.

**TABLE 1:** Frequency of appearance of gestures that accompany verbal speech in three learning cultures.

Codes	CL-R	CL-T	JZ-R	JZ-T	FL-R	FL-T
Play guitar without exemplifying purpose	2	0	0	3	35	13
Play guitar with exemplifying purpose	0	5	32	36	24	25
Gesture of playing guitar to set an example (without guitar)	14	12	0	5	0	0
Play guitar replacing verbal discourse	0	0	0	0	10	1
Foot kicks to support the verbal discourse	0	0	0	0	4	7

*CL, Classical apprentice; JZ, Jazz apprentice; FL, Flamenco apprentice; R, Reproductive apprentice; T, Transformative apprentice.*

### The Socio-Educational Context in Which Learning Cultures Are Inserted (Embedded Mind)

From the *ex post facto* study we have been able to observe some variables in common among the participants that outline the three learning cultures as embedded minds. The classical participants have completed high school studies and are in the process of university studies. Their specific musical studies are either undergraduate or in some cases postgraduate. Their families often include amateur musicians or music students. Up to seven of the jazz participants have university-level academic studies not necessarily musical. Their musical studies span twelve years or are equivalent to a professional degree in music, in the majority of formal classical studies that they have left. The study highlights that in their families there are usually no members who have had specific musical training. The apprentices of this culture are, on average, a decade older than the apprentices of the flamenco culture. Flamenco participants were educated to a maximum level of secondary school, most of them incomplete; with specific non-formal guitar musical studies of up to five years. They are very young apprentices who can already perform in public with a few years of specific training in the guitar. In all their families there are professional musicians. In flamenco culture, the perception that apprentices have of music cannot be separated from their family and social context, music is embedded in their way of life and therefore transcends musical activity itself, unlike the other two cultures where musical activity is more restricted to the context of musical production itself.

**FIGURE 3:**
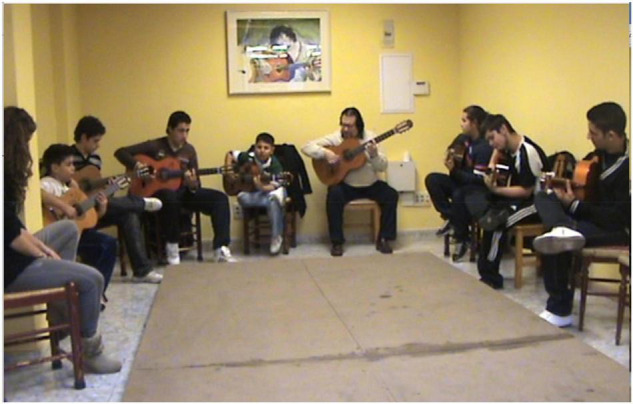
Daily group flamenco guitar class in the *Escuela del Maestro Entri* in Cañorroto, Carabanchel, Madrid, Spain.

We will now describe in-depth the flamenco guitar school and the two flamenco participants selected for this study, to gain a better understanding of their environment. It is important to point out that in the case of the school of master “Entri,” PS are daily and collective. All the guitarists go from Monday to Friday to group PS lasting approximately 2 h, in a large room and all the apprentice guitarists of all ages sit in a circle with the guitar in their hands (Figure 3). The room has a dance stage in the centre of the floor and is presided over by the teacher expertly playing the guitar. He also forms part of the circle, presiding over it and proposing routines and exercises which they then play either one by one until the circle is completed or all at the same time. Occasionally the teacher asks an advanced student to guide part of the session whilst he gives individual attention to a student in the adjacent room. Unlike what happens in other musical cultures (Casas-Mas, 2018), flamenco apprentices are embedded in a community of practice that has, however, a clear hierarchical organisation, from the teacher and advanced students to novices.

The first apprentice is 17 years old and a flamenco guitar student at the Academia de Caño Roto^5^ directed by the teacher, “maestro,” Aquilino Jiménez, “El Entri,” and both are of Roma ethnicity. The level of this student, described by his teacher, is advanced. He has studied with the teacher for four years and has already performed in public and professional events although he continues instrument training. He sometimes replaces the teacher in the group guitar classes in the school. He has finished his third year of statutory secondary education and the middle grade of a vocational training course in computing. He comes from a Roma ethnic family of music professionals, among whom is the teacher. His father plays the guitar on a regular basis in Evangelist church gatherings. We would classify his discourse as closest to traditional, repetitive learning.

The second apprentice is 15 years old and is a flamenco guitar student of the maestro José Jiménez, “el Viejín,” and both are of Roma ethnicity. For almost a year he was previously a student at the Academia de Caño Roto directed by the maestro Aquilino Jiménez, “El Entri.” He has performed at several public events as a guitarist, despite being very young at the time of this research. Since he was very young he has played the flamenco drum box. He comes from a Roma ethnic family with professional musicians, among them the maestro “Entri.” His grandfather sang fandangos in a professional show and his younger cousins also play instruments and sing. His father is pastor of the Evangelist church. He is studying the second year of statutory secondary education and intends to complete his education. He also likes blues and jazz music. His discourse is qualitatively different from the previous participant and is closer to constructive, comprehensive, and transformative learning.

During interviews apprentice, R never alludes at any time to learning issues in relation to other apprentices, but apprentice T mentions the possibility of practicing the piece with his peers as something that is easily within his reach and real. Therefore, this situation demands that all participating parties adjust to one another, those who are clapping, singing, or dancing, and those who are playing an instrument.

Practise the beat, if I say that to any girl [his sisters] come and get into the beat [plays], or to my cousins who are usually more often with me, they do not get tired because they probably like it as well. (T: Initial I.)

This is an indication of an element that is highly relevant for the understanding of the musical apprenticeship phenomenon in this culture but is expressed at a very low frequency in the individual discourse. No importance is attached to it because for them it does not fit into the “learning” category which we are asking questions about, it is understood as fun.

### Differences in the Activity of Learning Processes Within Flamenco Guitar Apprentices in the Oral Tradition (Enactive Mind)

Learning processes are neither passive nor reactive. They are the consequence of several neuronal connections that imply some kind of activity and these connections configure perception, emotion, attribution, movement, or the disposition to act (Noë, 2004). We will now describe specific elements of the individual PS of the two flamenco apprentices, that refer to this activity of the mind. The complete duration of the individual PS ranged between 6 and 19 min. They are brief sessions compared with academic cultures (that are around 60 min), since a great part of the practice takes place in a group setting, not individually. The main idea of the session was literal retrieval of the *falseta* that they had learned with their teachers. While the session of apprentice R was essentially reproductive with the creation of material just when the session was finished, the PS of apprentice T involved up to 12 interruptions of that literal retrieval in which the material played was totally new. The time distribution percentages of the PS of both apprentices are contained in Table 2.

**TABLE 2:** Dedication of time in percentages of the practice session of flamenco apprentices with Reproductive (R) approach, and with Transformative (T) approach.

Distribution of the PS	R (in minutes)	T (in minutes)
Literal retrieval and linking between fragments	97.4	80.9
No literal retrieval, adaptations instead	0	16.9
Creations with new material	2.6	2.2

**FIGURE 4:**
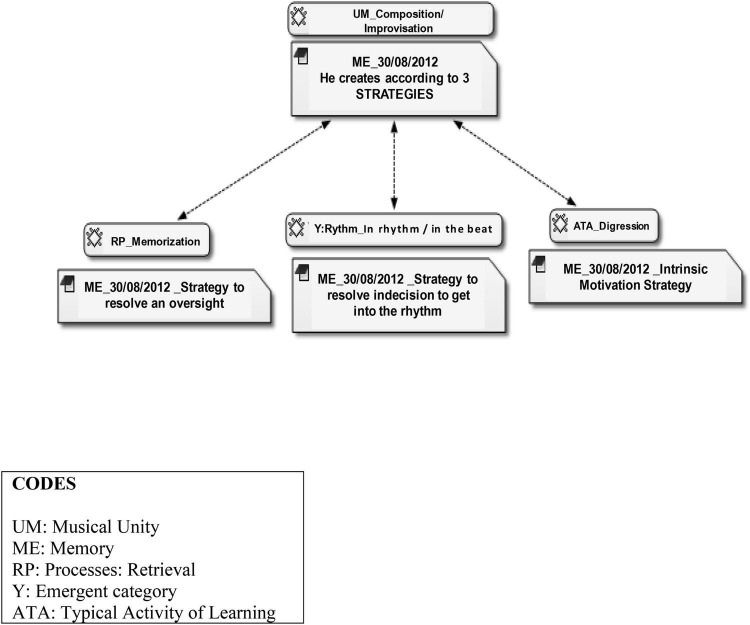
Sequence function where apprentice with Transformative approach establishes three strategies of musical creation.

The modification in literal retrieval in apprentice T was due to three types of reasons and each one, therefore, fulfils a different function (Figure 4). Firstly, the apprentice’s mobile where he had recorded the class with the teacher broke, so that he was unable to use it at home and could not retrieve the information properly. Under these circumstances, where memory is not entirely faithful, rather than stop the musical discourse the apprentice completed it with new information of his own. Secondly, the *falseta* started in an unconventional beat, the eighth of a total of twelve, which is difficult. Instead of always starting from silence the apprentice linked this *falseta* with rhythmic pattern sequences and a rhythmic *rasgueado* technique, as a “run-up” or by way of a filler. Thirdly, due to so many repetitions of the same material, the apprentice used his creations as a form of digression, i.e., without direct reference to the *falseta* in question, as an intrinsic motivation strategy to continue persisting in the task later.

We will now explain the arguments of this apprentice in the RPI in these three circumstances. In the first instance he was not concerned about having learned it in a different way to the original, or “with mistakes,” and this was not a worry to him at all. The second function was a strategy for playing a *falseta* starting on the eighth beat. Here self-regulation was detected from whispering to counting the beat, like the self-guidance described in detail in the categories which Casas-Mas et al. (2019) termed as *private singing* (Supplementary Video 2 in Supplementary Material). He used the numbering of beats to adjust the beginning of the *falseta*. This process differed from that of apprentice R, where we found the private signing was very internalised, emerging as a highly guttural whispering when confronted with difficulty, e.g., a negative assessment of what he was playing and the need to visualise the fragment again to try to reproduce it exactly as it was in the video. The third strategy was the use of digression as a source of intrinsic motivation strategy to continue persisting in the task later.

We observed a tentative relationship between the discourse of each apprentice and the way in which they practice, but it was truly surprising to find such differences in action, having selected the participants from their discursive production. There were, therefore, concordances between discourse and observation of posterior practice between participants. This represents the connection of activity between the verbal mind and the mind in procedural action (Casas-Mas, 2020). However, within each participant, it was striking that in the case of apprentice R we found there was incongruence between his discourse and his practice which we did not find in participant T. When we asked questions in the RPI apprentice R sometimes did not realise what had previously been practiced. For example, situations in which the researcher had observed that were possibly difficult for him, he denied knowledge of. The researcher then showed him one of these difficulties in the video recording asking for his opinion. This was when he reacted and played the fragment “properly,” but he did not acknowledge that it had been difficult in the past. On the contrary, for apprentice T there were no contradictions between discourse and practice. He easily recognised situations and actions he had not done “correctly.” His practice was ahead of or on a par with his discourse. He had automated strategies in his practice which he easily was aware of in the RPI. This difference between the reproductive and the transformative apprentice was also found in apprentices of other learning cultures (Casas-Mas, 2013).

### External Representations in Flamenco Learning of Oral Tradition (Extended Mind)

With regard to external representations, there are some outstanding features distinguishing the participants of flamenco culture from those of the classical culture, closer to the jazz culture. Here, the flamenco culture made use of temporary external representations, such as a drum machine to define the tempo and speed of typical *palos flamencos*, and videos, that were closely related to beat and rhythm, common in popular cultures. In jazz culture, for example, audio editing software (Band in a Box) and metronome are frequently used.

**FIGURE 5:**
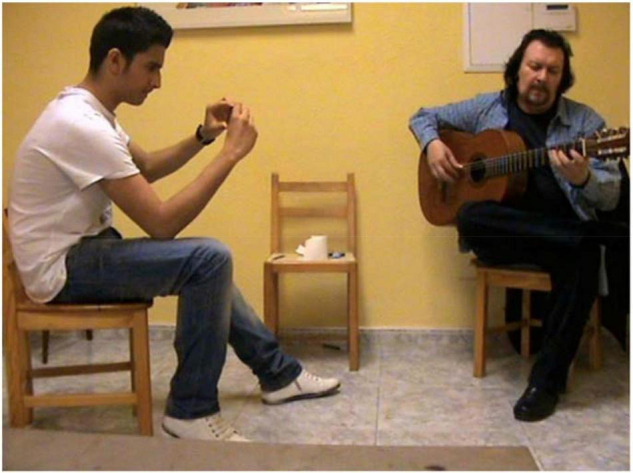
Apprentice Flamenco R records a video on his mobile of his teacher’s performance.

The apprentice R uses his mobile to record a video of the teacher (Figure 5) and then reproduces it to learn from it. The mobile phone functions as an extended mental resource that enables new cognitive functions, in this case as a mnemonic tool. In his own words, he describes the action of recording his teacher and reproducing it at home on this computer where the screen size is bigger for viewing with greater precision. The teacher makes a special version for recording characterized by lowering the usual speed of the *falseta* (Supplementary Video 3).

However, apprentice T describes getting knowledge mainly from listening. In his discourse, he stresses more what he hears than what he sees. Listening is his main source of knowledge and he associates it with repetition for generating a mental representation. He describes this representation as “having it in his head” and “getting the concept.” It is also an activity that intrinsically motivates him. This apprentice also says he uses the mobile for recording the teacher on video and then reproduces it to finish learning it. However, he specifies that the recording is support for long-term memory but the learning is done with the teacher on site. None of the apprentices, mentions listening to cover versions or any kind of notation since the latter is not used in oral tradition flamenco.

You have to have this in your head, you have to listen to it a lot […] I like getting out and inventing things […] (T: Initial I.)

The crucial difference between both apprentices is undoubtedly what they do with the auditory material. Whilst apprentice R only mentions the analytical processing, referring to technical guitar issues and pointing to certain sections of the piece, apprentice T also mentions the expressive and holistic processing. What we have called expressive processing refers to those procedural contents of an interpretative-intuitive nature that help us to embellish musical discourse. This is widely mentioned by this participant and is not linked to notation but to a large extent depends on the auditory appreciation. It is highly connected with considerations of rhythm, such as accents and entries or closures in certain specific beats.

We also found that it was very much linked to holistic processing, which is the one most characterised by this participant. In other words, with contents of an interpretative-intuitive nature, alluding to the holistic type comprehension or referring to the composer, style, and overall nature of the piece.

Tomate^6^ is pure rhythm. For example, Tomatito in all of his *falseta*s, you perform a Tomatito *falseta* and it won’t sound the same, because what he does with all of his notes is he tries to get in the rhythm, so it’s as if he is dancing all the time [plays an example]. The rhythm [beats with his foot] and the accents [beats with his foot], […] notice where it goes faster [plays], where the rhythm is held [plays], so that it comes out as if it were dancing [plays], you see? (T: Post-Practice I. 2)

The idea of creating applies both to the guitar performance and to other more global psychomotor procedures in the form of body extensions; like the dance that is another characteristic of their discourse and that he describes as something common to all people of the community:

[…] but I think that all [those of Roma ethnicity] create small choreographies, I mean any one of them you meet on the street [plays], anyone of Roma ethnicity you see and you say give us a *patada*^7^ and they will do one [plays], come what may. (T: Post-practice I.2)

Apprentice R gives us explicitly sung examples of what he is explaining in the verbal discourse on two occasions. The rest of the time we only observe guttural sounds accompanying verbal discourse and musical discourse, especially when he makes a mistake. However, apprentice T uses singing instead of using his guitar up to four times. He is an apprentice who integrates different modes of expression to communicate his musical idea. The mode of communication, such as dance, singing, or instrument interpretation, is only a means of this transmission, not an end. He explains that one of the difficulties he has found has been to focus on this expressive content of the *falseta*, and the solution he offers encompasses the previous ideas holistically.

[To resolve that difficulty] I play it by ear, like the teacher did, and I sing it to myself as if it was being danced. (T: Post-practice I.2)

## Discussion and Conclusion

The discussion will be presented by embracing the four objectives of this research and attending to the four factors that constitute the 4E Cognition in musical learning: Embodied, Embedded, Enactive, and Extended mind.

The first objective of this work was to observe the peculiarities of the body, posture, and gestures of advanced guitar learners comparing three cultures of learning, from formal to informal and with differences in musical literacy. The main conclusion is the importance of the body, verbal, and extralinguistic aspects in music learning, some of the main issues that the 4E highlights. This article allows us to conclude about the conformation of the embodied mind as a result of the culture of learning reflected through the body and the gesture in instrumental learning. In general terms, flamenco learners differed significantly from other cultures with a more sparing verbal speech (Baño, 2018; Casas-Mas, 2020), and compensated for this by greater use of facial and body gestures, as well as by playing the instrument constantly during their verbal communication. What is representative in them is a fusion between the body, music, and verbal discourse. We want to draw attention to emerging elements in relation to facial, eye, and eyebrow expression, as elements to be investigated in the future. The body eases the meaning-making process of learning music from early childhood (Nijs and Bremmer, 2019), and that process is the result of the limits that the body imposes on it, as well as the body being configured through its use. The posture of the flamenco and classical apprentices during practice was similar, no doubt due to their common origin. The crossed legs and closed-chest in the practice of these urban jazz participants might be hinted at as a reflection of greater introspection for creation. It could be the difference between drawing knowledge from within the individual or receiving it from without (Casas-Mas et al., 2015b). Although flamenco culture of learning is based on the literal transmission of musical knowledge between instrument teacher and student, we have been able to observe this fusion of communicative modalities, such as gestures, the body, the music, and verbal discourse, also in creative processes in other cultures (Besada et al., 2021). This multimodality has repercussions on their sense of identity or self, a state of mind of pre-reflective or embodied subjectivity (Høffding and Schiavio, 2021), and on the capacities or competencies that they integrate into their discourse, such as dance.

The second objective was to describe the environment where these apprentices develop, taking into account the relations they establish with teachers, family, and peers, or their *Embedded* minds. This is a brain-body system in continuous interplay with its niche where the organism enacts rich patterns of action and perception in a complex musical domain, as in some Roma communities, that dynamically unfold and shape each other, revealing new properties of a fertile environment that can be acted upon (Høffding and Schiavio, 2021). The study of “otherness,” as a culture of oral tradition, is relevant because of the learning richness it provides, and resources are needed for in-depth analysis of these communities from the inside. Previous research studies have already focused on socio-economically disadvantaged groups to prove that they have limited means of buying musical instruments and the repercussion this has had on their identity and enrolment in music conservatoires (Benedict et al., 2015; Burland, 2020). Furthermore, greater attention is increasingly given over to how indigenous musicians are overlooked by urban citizens, mass media, and even the national curriculum (e.g., O’Neill, 2009; Cain, 2015; Holguín and Shifres, 2015), coinciding with the analysis of the school texts by García Fernández et al. (2017) in Spain. This points out the barriers that Roma girls and boys face in their educational trajectory. It is equally the case that there are some descriptions of successful student models belonging to the Roma community (Abajo and Carrasco, 2004). Meanwhile, just as the “other” may be the flamenco apprentices compared with musical literacy apprentices, so may they also, and why not, be instrumentalist women in the area of this same learning culture. Incipient attention has been paid during the last decade to women flamenco guitar players (Cifredo, 2014; Recio Lineros, 2020), and the corporeity and roles of flamenco women in dance (Cruces Roldán, 2002, 2005, 2015) but much research remains to be completed in this field.

The third objective was to observe the differences in the speech and learning practice between flamenco guitarists selected as instrumental cases from the initial sample, with polarised approaches to learning. It was expected that observation would provide information on the activity or on the *Enactive* minds of the two apprentices, which could be differentiated according to their conception of learning and having an impact of this on their practice. This was another major factor for establishing such an in-depth qualitative study. Something in common is that both apprentices are highly motivated toward collective practice and classes with the teacher. However, solitary individual practice makes less sense and they are not very persistent in it. They show essentially hetero-regulated learning when they are working with the teacher, but very co-regulated learning when they are practising with peers, which is a huge part of their daily life, although they do not usually identify the latter as for learning but just for fun. Maybe because of that the discourse of both flamenco participants was usually focused on positive emotions, as found in learners of other cultures with a more constructive, or in this case transformative, profile (Casas-Mas et al., 2015b; López-Íñiguez and Pozo, 2016; López-Íñiguez and McPherson, 2020, 2021). We found there was a fair bit of similarity between these apprentices when observing the relationship between discourse and practice, in what they said they did and what they really did. In other words, the gap between explicit knowledge and more implicit or corporal knowledge may be lower than usual compared with learners of other cultures. Nevertheless, some differences between the two flamenco participants emerged during repetitive practice, when apprentice T was totally unfazed by having learned in a different way, “with mistakes” with regard to what the teacher had proposed, whilst R denied any difficulties or did not recognise any mistakes. We also have exemplified the perception of self-efficacy that apprentice T expresses when evaluating his own performance in the video recordings, coinciding with Zarza-Alzugaray et al. (2020).

The fourth objective refers to the use of symbolic or specific tools as *Extended* mind. With the flamenco community of oral tradition, it would not make sense to consider the mind extended through scores, or the theoretical mind (Donald, 1991), but rather the mind extended through gestures, or the mimetic mind (Goldin-Meadow, 2003) and the different levels of explicitness or abstraction that it implies. In the discourse on both flamenco apprentices, they emphasised playing by ear, although both appreciated the image and recordings in the video, mentioning how they greatly focused on the sound. Nevertheless, the crucial difference is that whilst apprentice R only mentioned analytical type elements or technical issues of the guitar in certain sections of the piece, T also mentioned contents of an interpretative-intuitive nature which alluded to a holistic type of comprehension, like dance or movements, or those referring to the emotion or the style and global nature of the piece, related to a multimodal explicit conception. The possibility of learning from video recording has an impact on the activation of learning processes has been observed in previous research (Boucher et al., 2020; Volioti and Williamon, 2021) and can contribute to important implications for music education in both formal and informal learning.

The gesture is always linked to the context of production; that is why these apprentices when interviewed sing music more than talk about it. This affects self-regulation processes using private speech or singing during learning, coinciding with that defended by McPherson and Gabrielsson (2002) regarding the primacy of auditory over the symbolic-notational image, these apprentices construct only the auditory image. However, we observed that they do not specify the melody, neither by singing, humming, or whistling that we saw in other cultures (Casas-Mas et al., 2019). We only detected very guttural sounds in apprentice R and whispering of counting beats as self-guidance in apprentice T. This suggests a profound internalisation of private speech and song, coinciding with that observed in other popular cultures, with less use of notational material. The body provides us with a lot of information about these learners of oral tradition and paves the way for future exploration in a field that continues generating interest in the area of educational psychology (Mulvihill et al., 2020).

Our main conclusion is, as Schiavio et al. (2021) were emphasising, that there is a need to examine within ecologically valid settings, how musical ability, e.g., to correctly synchronise, emerges and flourishes in musically rich contexts like the flamenco community, where the rhythm and the beat may take years to develop. The flamenco players who participated on this occasion were learning guitar with a teacher but prior to this, they had already learned music as a mother tongue since infancy, as described by Lave (2011), Rogoff (2012) in other domains. It is as if these flamenco apprentices needed the perception of a participating audience in order to practice music in a process of participatory sense-making (De Jaegher and Di Paolo, 2007; Krueger, 2014). In this realm singing and dance were not contradictory with playing an instrument but were even taught and lauded from the social environment, as an actual lived experience (Reybrouck, 2021). This is different from learning music as a second language, i.e., symbolising with musical signs, decoding the meaning of the musical score, and coding the material again into musical productions, just as the formation of musical language continues prioritising in the Western classical tradition and in contexts, such as the conservatory.

The limitations of this work are that the findings come from a small number of participants and therefore cannot be immediately generalised to other apprentices or cultures of learning. However, this method provides information on situated learning processes that are explicitly verbal first hand but are also implicitly informed due to its focus on embodied knowledge. Some regularities between learning cultures, and a detailed description that had not been previously made are the main contributions, that may open possibilities for future lines of research in other cultures or focusing on external representations from the 4E Cognition approach.

## Data Availability Statement

The original contributions presented in the study are included in the article/Supplementary Material, further inquiries can be directed to the corresponding author/s.

## Ethics Statement

The studies involving human participants were reviewed and approved by the Autonomous University of Madrid. Written informed consent to participate in this study was provided by the participants’ legal guardian/next of kin. Written informed consent was obtained from the individual(s), and minor(s)’ legal guardian/next of kin, for the publication of any potentially identifiable images or data included in this article.

## Author Contributions

AC-M organized the database and wrote the first draft of the manuscript. All authors performed the analysis, and contributed to the conception, design of the study, and manuscript revision, read, and approved the submitted version.

## Conflict of Interest

The authors declare that the research was conducted in the absence of any commercial or financial relationships that could be construed as a potential conflict of interest.

## Publisher’s Note

All claims expressed in this article are solely those of the authors and do not necessarily represent those of their affiliated organizations, or those of the publisher, the editors and the reviewers. Any product that may be evaluated in this article, or claim that may be made by its manufacturer, is not guaranteed or endorsed by the publisher.

## Footnotes

1 *Payo* is the name that members of the Roma community assign to people who do not belong to it.

2 Dictionary of the Royal Academy of the Spanish Language, the fifth meaning of Gitano (Roma): http://lema.rae.es/drae/?val = gitano (January 2022).

3 The *falseta* is a fragment of music in the complete sense that it has a beginning, middle and end. Duration is variable, and it is used as a combinable module with other musical sections which adapt well in rhythm, speed and tone. The *falsetas* are pieces taught by teachers to students, or between equals. Many are the creation of the emblematic maestros themselves and others already form part of the tradition. In some cases, they are composed by the actual apprentices.

4 Osmosis is a term taken from biology that defines a reciprocal influence between two individuals or elements that are in contact. In this case the contact is metaphorical.

5 Caño Roto is an area belonging to the Latina district, in the south of the Madrid capital of Spain. It is part of a social housing project carried out from the 1950s to bring order to spontaneous shantytown settlements. An extremely high percentage of citizens residing in this neighbourhood did not finish primary education and it occupies the fifteenth place in the income level of the capital (Government Area of Economy and Employment, 2012).

6 *Tomate* refers to the guitar master Juan Manuel Muñoz who was born in Córdoba in 1970 and was the son of the mythical guitarist Juan Muñoz, who was also nicknamed “Tomate” and brother of the three flamenco-pop singers “las Ketchup,” who popularised the song *Aserejé* in 2002.

7 *Patada de baile* is a small choreography that involves body movement and footwork, which each person adapts, composes or improvises in their own way, so they are often valued for their creativity and flow as much as for their technical prowess. It usually has a duration of musical phrase according to the different rhythms or *palos*.
