# Supplementary material for: Oral Tradition as Context for Learning Music From 4E Cognition Compared With Literacy Cultures. Case Studies of Flamenco Guitar Apprenticeship
Source: Front Psychol. 2022 Apr 29;13:733615. doi: 10.3389/fpsyg.2022.733615 (PMC9106530; doi:10.3389/fpsyg.2022.733615)
Supplement: Supplementary file 1 [file Data_Sheet_1.pdf]

UAM
UNIVERSIDAD AUTONOMA
DE MADRID

**Participant:**	**Age:**	**Date:**	**Code:**
**Tlf:** **e-mail:**			
**Musical piece:**			
**Initial Interview**			

- Why have you selected this theme musical piece?

- How long do you think it will take you to prepare it to play it in a concert?

- We are going to divide that time into three moments: beginning (today), middle and end of the process (just before playing it in a concert).

1st session (date):

2nd session (approximate date):

3rd session (approximate date):

With this theme (piece, work), how do you start to practice (study)?

What do you think will have happened when you get to the next session (2nd Practice Session)?

What do you want to learn in that time?

What do you think will have happened when you get to the last session (3rd Practice Session)?

What do you want to learn in that time?

UAM
UNIVERSIDAD AUTONOMA
DE MADRID

**Participant:**	**Age:**	**Date:**	**Code:**
**Tlf:**	**e-mail:**		

POST-PRACTICE INTERVIEW

- When you picked up this piece at the beginning, what did you think was the most important thing you had to learn to know this piece? (5 apprenticeships approx.)

1.
2.
3.
4.
5.

- Which do you think is the most important? ---
- What difficulties have you found in this learning phase?

1.
2.
3.
4.
5.

- How have you solved/would solve each one?

1.
2.
3.
4.
5.

- How satisfied are you with that session?
